# LOWER BACK PAIN AFFECTS MOBILITY-RELATED INJURIES IN OLDER ADULTS

**DOI:** 10.1093/geroni/igz038.1071

**Published:** 2019-11-08

**Authors:** Tyler R Bell, Pariya Fazeli, Caitlin N Pope, Michael Crowe, Karlene Ball

**Affiliations:** 1 Pennsylvania State University, State College, Pennsylvania, United States; 2 University of Alabama at Birmingham, Birmingham, Alabama, United States; 3 Center for Injury Research and Policy, The Research Institute at Nationwide Children’s Hospital, Columbus, Ohio, United States

## Abstract

Pain causes functional limitations and might elevate risk for mobility-related injuries in older adults. For this reason, we examined the longitudinal impact of lower back pain on the likelihood of MVCs and falls. Between 1998 and 1999, participants (ages >55 years) completed cognitive and physical measures at three Motor Vehicle departments. Participants then completed a telephone interview (n=1,248) assessing yearly health complications and injuries, which continued annually for 14 years. Separate longitudinal models examined the relationship between lower back pain and MVC and fall likelihood while controlling for demographics and mobility. Overall, those with lower back pain were twice as likely to have a fall than unafflicted peers (95%CI:1.69-2.47) and odds of MVC was just beyond statistical significance (95%CI: 0.97-1.94). In persons with lower back pain, problems in lower-limb function, divided attention, and task-switching were associated with MVCs whereas problems in lower-limb function were related to falls. Addressing limitations from pain might reduce mobility-related injury in older adults.

